# Playing a cooperative game promotes preschoolers’ sharing with third-parties, but not social inclusion

**DOI:** 10.1371/journal.pone.0221092

**Published:** 2019-08-19

**Authors:** Theo Toppe, Susanne Hardecker, Daniel B. M. Haun

**Affiliations:** 1 Department of Early Child Development and Culture, Leipzig University, Leipzig, Germany; 2 Leipzig Research Center for Early Child Development, Leipzig, Germany; 3 Max Planck Institute for Evolutionary Anthropology, Leipzig, Germany; 4 SRH University of Applied Health Sciences, Gera, Germany; Universidad Loyola Andalucia Cordoba, SPAIN

## Abstract

This study examined the effect of gaming context on young children’s prosocial behaviors. Dyads of 4- to 5-year-old children (*N* = 96) played the same game cooperatively, competitively, or solitarily. After playing the game for a total of ten minutes, sharing with and social inclusion of uninvolved third-parties as well as free play with previous co-players was observed. Children shared less with third-parties after playing the game competitively than after playing it cooperatively. Playing a solitary game resulted in intermediate levels of sharing. The structure of the game did not differentially impact measures of social inclusion or free play.

## Introduction

Preschoolers spend a substantial amount of their time playing games [1]. Doing so helps them to acquire motor skills and facilitates their socio-cognitive development [2,3]. Furthermore, games can promote both adult-to-child- and child-to-child-transmission of moral values [2,4,5]. To learn more about the acquisition of moral values via games, we investigated how games influence different aspects of preschoolers’ prosocial behaviors—acts intended to benefit others [6].

### Social Interdependence Theory

Games differ in diverse characteristics such as content, determination of outcome (e.g., strategy, chance, or dexterity), and context [7,8]. The characteristic that describes the essential social dynamics of a game and probably influences children’s social behavior substantially is the gaming context. Gaming context refers to the relation of players’ goals in a game. Based on Social Interdependence Theory (SIT; [9]), three gaming contexts can be derived: Cooperative, competitive, and solitary. Cooperative games are characterized by a positive interdependence (i.e., a positive relation between the players’ goals): Players win and lose together. In competitive games, players’ goals have a negative interdependence: One player’s victory leads to the co-player’s loss and vice versa. When playing solitary games, players’ goals are independent, meaning that success or failure of one player does not affect the results of the co-players.

According to the SIT, the relationship between agents’ goals has an impact on their social behavior [9]. Positive interdependence results in a cooperative mindset that is characterized by the anticipation of help, a benevolent attitude toward interaction partners, and an egalitarian morality [10]. Negative interdependence elicits contrary orientations characterized by the anticipation of resistance, aggression, and a preference for advantageous inequality [11]. The absence of any interdependence neither promote nor hamper any of these attitudes [12]. Importantly, SIT claims that recipients generalize positive or negative actions of an agent to the agent as a whole [12,13], thus resulting in carry-over effects to new situations. A second assumption is that “effects elicited by a given social relationship also tend to elicit that type of social relationship” ([14], p.30). This implies that cooperative actions of agents promote cooperative actions of their co-players. Corresponding relations are assumed for competitive and solitary behaviors.

### Games and prosociality

A large body of evidence supports the effects of cooperative, competitive, and solitary games on children’s prosociality proposed above. *While* playing cooperative games, preschoolers behave more cooperatively [15,16] and show more prosocial acts [17,18] as compared to playing competitively. When playing cooperative games first-grades use more group-oriented statements [19] and high-level negotiation strategies [20] as compared to when playing competitive games. Additionally, preschoolers behave more aggressively while playing competitive games as opposed to playing cooperative games [15].

Not only during the gaming situation itself, but also *after* playing cooperative games, preschoolers show higher levels of cooperation in free play in comparison to competitive games [15]. Children initiate more positive physical contact [16,21,22] and more positive cross-ethnic interactions with their co-players after playing cooperative games [23]. Cooperative gaming interventions reduce subsequent aggressive behaviors [15] and promote subsequent altruism [24–26] in 4- to 7-year-olds. Recent experimental work shows that collaboration has a crucial effect on children’s prosociality. After working collaboratively, preschoolers share the rewards more equitably as compared to after working solitarily [27,28]. This effect seems to be due to a promoted sense of fairness rather than a general increase in children’s generosity [29]. Crucially, in these experimental studies, resources were obtained through collaborative or solitary activities and the distribution of resources was a part of the activity itself. Yet, 5-year-olds even shared fewer resources that were unrelated to competition (i.e., stickers in a coloring contest) with competitors as compared to third-parties [30]. Further, preschoolers helped, trusted, and liked partners with whom they experienced collaboration more than neutral others [31].

Taken together, cooperative, competitive, and solitary games and activities substantially impact co-players’ interactions. But do cooperative games in contrast to competitive and solitary games also promote prosociality toward uninvolved third-parties (i.e., non-players)? To address this question, Orlick [32] implemented an 18-week gaming program to explore the effect of cooperative and solitary games on sharing behavior of 5-year-olds. Compared to a baseline, children shared more stickers with an anonymous peer after playing cooperatively but shared less in the solitary condition. Street and colleagues [33] assessed the effect of a 3-month cooperative gaming intervention on the prosocial behavior of 9- to 12-year-olds. Children in the intervention group played cooperative games biweekly, while participants in the control condition received regular physical education. After the intervention, parents and teachers rated participants in the intervention group as generally more prosocial compared to participants in the control group. Corresponding evidence comes from Battistich and colleagues [34], who compared two groups of elementary students (5-year intervention group vs. a control group receiving regular education) in their usage of prosocial strategies in hypothetical conflict situations. Amongst others, the intervention program included cooperative tasks, sessions highlighting prosocial values, and helping activities (e.g., peer tutoring). In interviews, children described their hypothetical social problem-solving reactions in story vignettes (e.g., one child takes away a toy from another). Importantly, participants were asked to imagine themselves being the disadvantaged agent in the narrated story. Results indicated a higher usage of prosocial strategies and more consideration of others’ needs in the experimental group as compared to the control group.

In sum, existing evidence shows that in contrast to competitive and solitary games, cooperative games can foster prosociality toward co-players and third-parties who did not participate in the game. However, existing research suffers from three major drawbacks. The first and most important drawback is the low comparability between cooperative and control conditions. The previously games used do not only differ in their context, but also in the behavior being reinforced (e.g., experimenter reinforce prosocial behaviors in cooperative, but not in competitive or solitary conditions), the content of the game, and the difficulty of the task [15,24,26,32,33,35]. Second, no prior study has systematically compared the impact of the same game played across all three gaming contexts. This comparison is necessary to ascertain whether cooperative games foster prosocial behavior in contrast to both solitary and competitive games, competitive games impede prosocial behavior as compared to solitary and cooperative games, or whether both processes work together. Third, existing evidence fails to assess and control for the gaming performance (i.e., winning or losing) as a predictor for subsequent prosociality. Gaming performances might influence children’s mood, which has been found to impact their prosociality [36]. Therefore, gaming performance might have an impact on subsequent prosociality and should thus be considered as a potential mechanism. To overcome these shortcomings, the present study (i) uses the same game for all experimental conditions, (ii) compares all three gaming contexts, and (iii) includes gaming performance as a predictor for subsequent prosociality. Additionally, this is the first study addressing short-term effects of a cooperative gaming intervention in children. The investigation of short-term effects will give us further insight into the learning process underlying long-term gaming interventions.

### The current study

To investigate how different gaming contexts influence preschoolers’ prosociality toward co-players and third parties, we created a novel game that can be played cooperatively, competitively, and solitarily. In the game two preschoolers need to navigate marbles into holes in order to score. Dyads of 4- to 5-year-old children played this game in one of three contexts (between-dyad-design). We assessed 4- to 5-year-olds, because children at this age have developed an understanding of cooperative and competitive game structures [37]. After playing the game, three measures of prosociality were assessed: Sharing, social inclusion, and free play behavior. Sharing was measured by a dictator game, in which children could divide an endowment of ten stickers between themselves and an unfamiliar absent third-party (same-sex peer). Social inclusion was assessed using a novel task, in which children could include a third-party into an ongoing ball-tossing game. Finally, we observed co-players during free play and coded their playing context and prosociality. We preregistered our study (https://osf.io/y4dk7) and indicated deviations from the preregistration. Detailed procedure, materials, coding sheets, analysis script, and data have been made publicly available on the Open Science Framework (https://osf.io/jasbz/).

We predicted that playing a cooperative game would promote preschoolers’ post-game sharing with and social inclusion of third-parties as compared to playing a competitive and solitary game. For free play, we predicted that after playing a cooperative game, children would show more prosocial acts toward their previous co-player as compared to the other conditions. In addition, we predicted that children would transfer the gaming context to free play (i.e., more cooperative play after playing a cooperative game, more competitive play after playing a competitive game, and more solitary play after playing a solitary game).

## Materials and methods

### Participants

In total, 96 children in 48 unfamiliar, same-sex, and same-age dyads participated in the study. Children were between 4 and 5 years of age (*M* = 5.03 years, *SD* = .59, range = 4.02 to 6.00), came from diverse socioeconomic backgrounds, attended kindergarten in a medium-sized German city, and were recruited from a laboratory-maintained database. A power analysis recommended a sample size of 126 to 133 participants (see preregistration). Due to personnel and time constraints, we decided to test 96 children. Our study might be slightly underpowered which might impede the interpretation of our results (see Discussion). The ethics committee of the Medical Faculty of Leipzig University approved the study (approval number 169/17-ek). Prior to testing, parents gave informed consent for their children’s participation. Dyads were tested in a laboratory in sessions lasting approximately 40 minutes. Data collection took place between June and November 2017. Eighteen additional dyads were tested but excluded from analyses due to children’s reluctance to participate (*n* = 12), participants’ acquaintance (*n* = 2) or experimenter error (*n* = 4). To be included, subjects needed to have valid values for at least two out of three outcome variables (sharing, social inclusion, and free play). For the majority of the sample (88.5%) all three outcomes were available. Number of missing values were five for sharing, two for social inclusion, and four (two dyads) for free play.

### Materials

**Fig 1:**
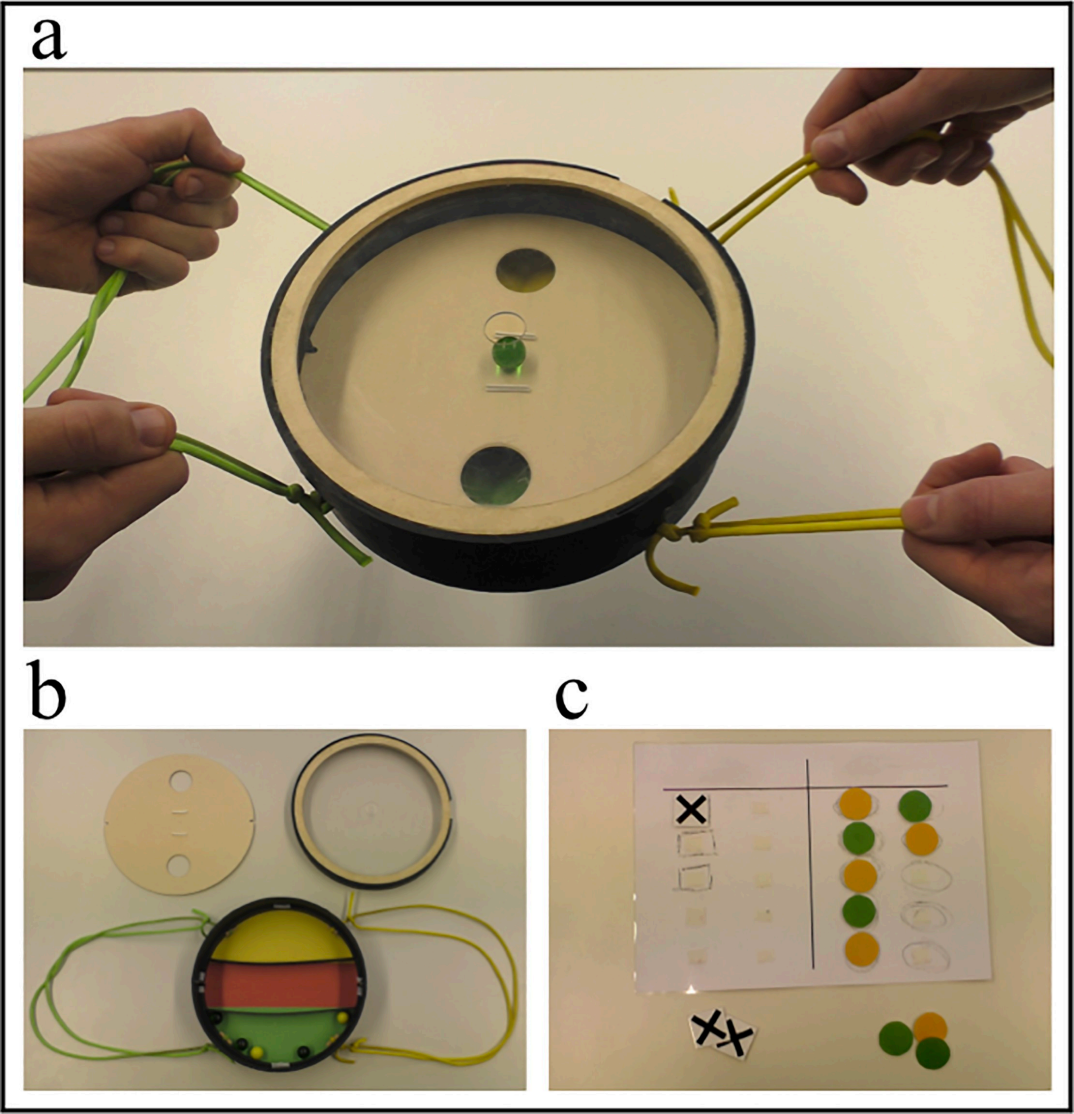
Material for gaming phases. Figure depicts (a) apparatus as played by the children, (b) deconstructed gaming apparatus for illustration, and (c) scoreboard, points, and crosses.

Children played a game “KoKo”, in which they needed to navigate colored marbles into holes on a round platform (Fig 1A). The round case (diameter approx. 23cm) and platform with two holes were made of dashboard. Marbles which fall into a hole are caught in a storage under the platform (Fig 1B). The base plate is colored either green or yellow under each hole. A Plexiglas lid with a hole in the center for placing marbles on the platform was used to prevent marbles from falling out the case. Each player can navigate the marbles by tilting the platform with two ropes colored green and yellow. Marbles had a diameter of 16mm and were colored green and yellow. Additional material for the game was a laminated scoreboard, green and yellow dots and black crosses (Fig 1C). We had a second identical game colored red and blue for the solitary condition.

**Fig 2:**
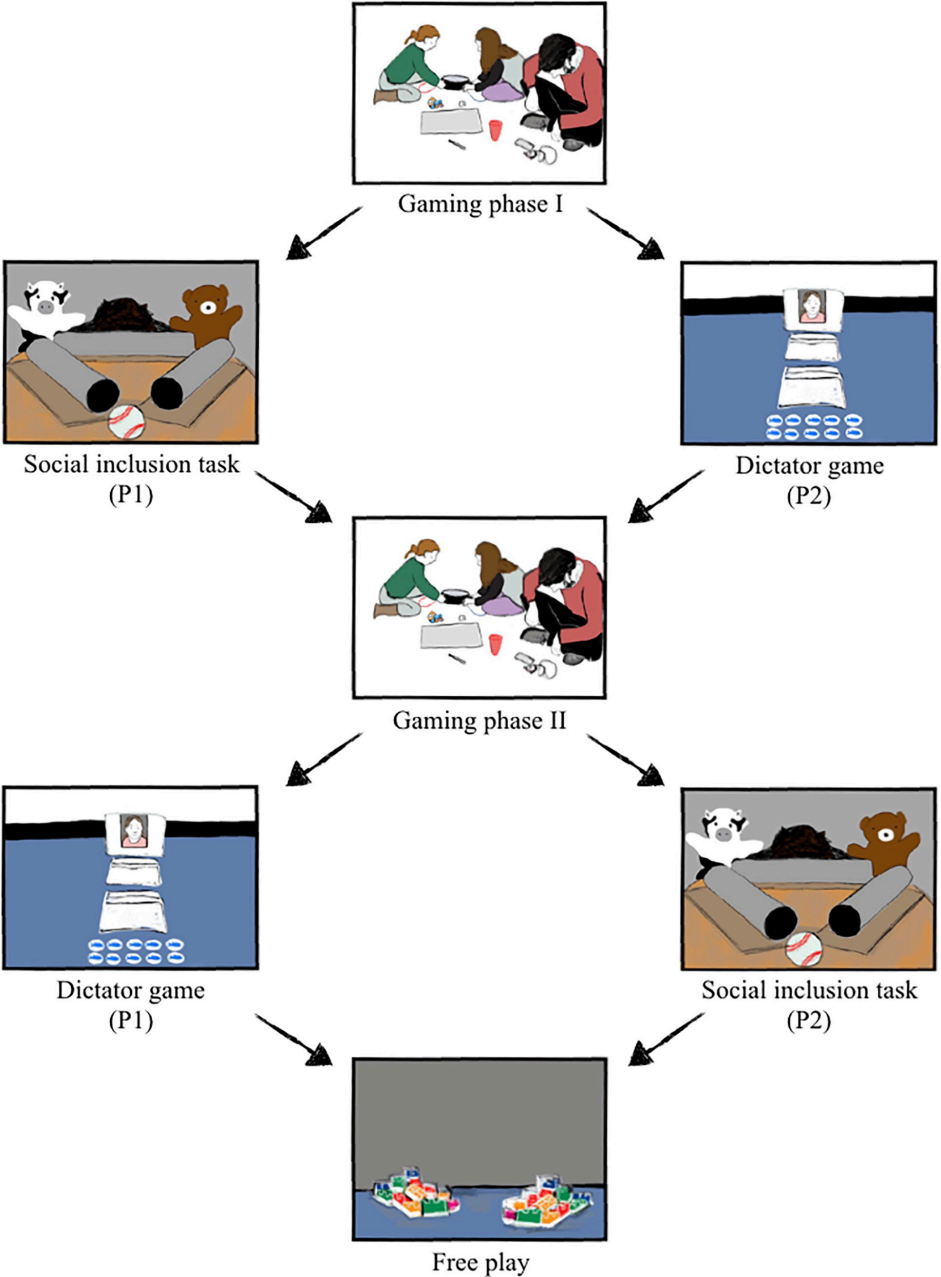
Experimental procedure for both participants. Participants experienced gaming phase I, gaming phase II, and free play together. Participant 1 (P1) first completed the social inclusion task and then the dictator game, while participant 2 first completed the dictator game and then the social inclusion task. The dictator games and inclusion tasks were tested in separate rooms.

Stimuli for the dictator game were portraits depicting a boy or a girl (matching the participant’s sex) with happy facial expressions. Pictures were taken from the NIMH Child Emotional Faces Picture Set [38]. Participants were provided with ten identical stickers. In the social inclusion task, we used a triangle of opaque fiberglass tubes which were fixated on a wooden frame (Fig 2). Tubes were approximately 50cm long and had a diameter of 8cm, so that a rubber ball (diameter approx. 6cm) could easily fit through them. Additionally, two animal hand puppets (a bear and a cow) were used in this task. For the free play, we used 40 enlarged toy blocks.

### Design and procedure

The study design comprised three between-dyad conditions: Cooperative (*n* = 32; 16 female), competitive (*n* = 32; 16 female), and solitary (*n* = 32; 16 female). Dyads were randomly assigned to one condition. Dependent measures were sharing with and social inclusion of a third-party, as well as playing context and prosociality in free play with co-players. Two experimenters conducted the study; Experimenter 1 (E1) conducted the gaming phases, the social inclusion task and free play, while Experimenter 2 (E2; blind to condition and hypotheses) conducted the dictator game. Before testing, children were randomly assigned to either the role of participant 1 (P1), who did the social inclusion task first followed by the dictator game, and participant 2 (P2), who completed the tasks in reversed order (detailed description below).

Procedure started after a warm-up phase, in which the direct interaction of the participants was limited by the experimenters. Throughout the experiment participants played KoKo two times for 5 minutes each (Fig 2). After the first gaming phase, the dictator game and social inclusion task were conducted separately, meaning that P1 first did the social inclusion task and P2 played the dictator game. Hereafter, both participants played KoKo again in the same condition. Subsequently participants changed roles, meaning that P1 participated in the dictator game and P2 in the social inclusion task. Afterwards, both participants played freely for 5 minutes.

#### Gaming phase I

The procedure started with a gaming phase of KoKo. In each experimental condition, E1 introduced KoKo with different rules. In the cooperative condition, both participants had the same goal and mutually needed to navigate colored marbles into correspondingly colored holes on the platform. If participants scored, they jointly received a point on the scoreboard. If participants navigated the marble into the wrong hole they received a cross. If participants scored 10 points, they won a round together. If they received 3 crosses, dyads lost a round. In the competitive condition, participants had contrary goals. P1 needed to get a neutrally colored marble into the yellow hole, while P2 had to bring the same marble into the green hole. By scoring, participants individually received points. The participant that scored 5 points first won a round, whereas the other lost. In the solitary condition, participants each had their own gaming apparatuses. Both participants needed to maneuver colored marbles into corresponding holes. If participants scored, they received a point on their own scoreboard. If they failed, participants received a cross on their own scoreboard. Participants won a round if they scored 10 points. When they receive 3 crosses, they lost a round. In this condition, participants played the game in parallel in the same room, standing back to back, and were instructed to focus on their own game to prevent comparisons of the players’ results and possible competition, which occurred occasionally.

In each condition, gaming duration was approximately 5 minutes (*M* = 308.21s, *SD* = 6.18, range = 292 to 328). E1 managed scoreboards and supplied participants with new marbles. If participants completed a round within the gaming time, E1 immediately started a new round by clearing the scoreboard and supplying a new marble. Before playing the game, participants were asked three questions to check their comprehension of the game: Where they needed to get the marbles to score; what happened if they scored and failed; to whom the dots and crosses on the scoreboard belonged. If participants did not answer one of the three questions correctly, E1 repeated the relevant information and asked the participant again. All dyads passed the comprehension check either spontaneously or after one repetition. After the first gaming phase, P2 was guided to a separate room, in which E2 waited. P1 stayed in the test room with E1.

#### Dictator game

E2 conducted a dictator game with P2. Our procedure was based on previous studies that successfully used this method in preschoolers (e.g.,[39]). Participants got an endowment of 10 identical stickers and had the opportunity to share a self-chosen amount of these with an absent and unfamiliar peer. Importantly, stickers were not related to the game. The unfamiliar peer was introduced as a same-sex child, who would come to the laboratory the next day. Participants could share stickers by placing them in an envelope lying in front of a picture showing the unfamiliar child. Stickers that participants wanted to keep for themselves could be placed in a second envelope close to the participant. While placing the stickers, E2 turned around and did not observe the participant. To ensure that participants understood the instruction, they were asked four questions before dividing the stickers. They were asked to whom the stickers belonged; where they could place the stickers they want to dispense; where they could place stickers they want to keep for themselves; whether anyone could see them, while placing the stickers. The instruction was repeated if one of the questions was answered incorrectly. Most children (60.7%) passed the comprehension check spontaneously and all after one repetition.

#### Social inclusion task

Simultaneously, E1 conducted the social inclusion task with P1. To measure social inclusion behavior, a ball-tossing game was introduced. In the beginning of the tossing game, participants played the ball back and forth through each tube with a puppet operated by E1 (depicting a bear; hereinafter ‘familiar puppet’). Within this familiarization, the familiar puppet introduced a second ‘unfamiliar puppet’ (depicting a cow) sleeping beside the apparatus by stating: “Look! There is someone sleeping. But we can continue to play.”. After one round of passing the ball through each tube, the familiar puppet stayed at one corner of the apparatus (counterbalanced) and initiated another two passes to the participant. When the familiar puppet held the ball, the unfamiliar puppet (also operated by E1) suddenly appeared at the vacant corner of the triangle stating “Hello”. The familiar puppet decided to pass the ball to the participant and not to the approaching unfamiliar puppet after looking at both tubes and thinking aloud about where to pass the ball by stating “Do I pass the ball to the cow or to [Name of child]?”.

Then participants could decide to which puppet they wanted to pass the ball. If any puppet received the ball, the puppet happily stated that the ball arrived and passed the ball back to the child. Puppets did not pass the ball to each other. If not included for two consecutive passes, the unfamiliar puppet gave standardized prompts, indicating the desire to join the play. Prompts were as follows: “Hello, I am the cow.”, “Can I join you?”, “Could you pass the ball to me?”, “Pass the ball to me!”. Subsequent to each prompt the familiar puppet again decided to pass the ball to the participant and not to the unfamiliar puppet after thinking aloud about to whom to pass. Ten passes of the participant (and 10 returns of the puppets) with these rules were followed by a forced-choice trial, in which the familiar puppet asked participants to whom it should pass the ball (to the unfamiliar puppet or to the participants themselves). For nine subjects the number of passes was not 10 due to experimenter error (range 9 to 12 passes). Deviating from our preregistration, we decided to include these subjects into the data analysis, since all subjects with only 9 passes already included the unfamiliar puppet before the error took place. For the calculation of the inclusion ratio (see below) we considered all passes played by these subjects.

#### Gaming phase II and role change

After being tested separately, both participants played KoKo for another 5 minutes. E1 reviewed the rules of the game and did a comprehension check as described earlier. Importantly, gaming context was the same as in the first gaming session, but participants changed colors (or apparatuses in solitary condition). Hereafter, the dictator game was conducted with P1 and the social inclusion task with P2, to assess sharing and social inclusion for both participants.

#### Free play

Finally, free play of both participants was observed. After waiting for E1 to prepare the test room, participants played with enlarged toy blocks placed in two bunches (20 blocks each) at the opposite ends of the room. E1 guided the participants into the room and said: “Look! There are toy blocks and the two of you can play with these!”. Then E1 left the room and participants could freely play for 5 minutes. After the free play session the procedure ended and children were awarded with a small gift for their participation.

### Coding

All sessions were videotaped (two gaming sessions were not recorded but duration and result were coded live). Coding was done live and from video by the first author. We coded gaming performance for each participant by dividing participants’ points by the number of all marbles played. In addition to our preregistration, we coded the number of rounds that participants won and lost and calculated the difference between these. We decided to use the difference of the number of lost rounds from the number of won rounds as operationalization of gaming performance. We did so since the described ratio of points and total marbles is difficult to compare between conditions and does not take winning into account. The difficulty of comparing the three conditions arises from the different scoring systems. In the cooperative and solitary condition, children needed 10 points to be successful and three crosses entailed a loss, whereas in the competitive condition 5 points lead to a victory over the co-player. Consequently, the mean ratio of points and totally played marbles is always .50 in the competitive condition, since a marble is a benefit for one player and a misfortune for the other. This is not the case in the cooperative and solitary condition. The difference between won and lost rounds seems to be a more suitable proxy for the gaming performance, because not only quantity (number of points), but also quality (winning and losing) are considered.

For the dictator game, we coded the number of shared stickers. For the social inclusion task, we coded whether participants included the approaching unfamiliar puppet within all 10 passes or not (hereinafter *general inclusion*), with which pass participants included the unfamiliar puppet the first time (hereinafter *first inclusion*), and the chosen option in the forced-choice trial. Additionally, we calculated an *inclusion ratio* of the passes to the unfamiliar puppet divided by all passes after the first inclusion. A higher ratio indicates more passes to the unfamiliar puppet. For each participant, free play was coded with regard to gaming context and the number of prosocial behaviors. For the coding of gaming context, playing sessions were segmented in 10s intervals. Gaming context could either be cooperative, competitive, or solitary. Play was coded as cooperative if participants asked for or attained the same goals (e.g., building something together). Play was coded as competitive if participants attained opposite goals (e.g., contests or stealing blocks from one another). Play was coded as solitary if a participant played independently (e.g., building towers alone). We also coded if a participant did not play (e.g., ignoring toy blocks). Sharing, helping, and comforting were considered as prosocial behaviors [40].

### Reliability

Independent coders blind to hypotheses coded 25 percent of the data of all dependent and control variables. We calculated Cohen’s *κ* for variables with nominal scales and intraclass correlation coefficient (*ICC;* [41]) for metric scales. Interrater agreement for the ratio of points to total marbles (first gaming phase *ICC* = .97; second gaming phase *ICC* = 1.00) and the difference between won and lost rounds (for first gaming phase *ICC* = .98; for second gaming phase *ICC* = .97) was perfect. Interrater reliability was excellent for the dictator game (*ICC* = 1.0), general inclusion (Cohen’s *κ =* 1.0), first inclusion *(ICC =* 1.0), inclusion ratio (*ICC =* 1.0), chosen option in the forced-choice trial (*κ =* 1.0), and the number of prosocial behaviors in free play (*ICC* = .75). Reliability for the gaming context of free play was substantial (Cohen’s *κ =* .68).

### Data analyses

Our main question was whether gaming context has an effect on preschoolers’ sharing with, social inclusion of third-parties, and free play with co-players. To address this, we analyzed our data using generalized linear mixed models (GLMM) for sharing, social inclusion, and number of prosocial behaviors in free play. We included experimental condition, age in days, the interaction of both, gaming performance of the phase immediately played before (difference between number of rounds won and lost), and sex as fixed effects in all models. For prosociality in free play, which always followed two gaming phases, we included the mean of both gaming performances. Dyad identification number was added as a random intercept effect. For each dependent variable, we firstly compared the fit of the full model with the fit of a null model, containing only the random intercept effect and sex by using a likelihood ratio test. In case of a significance, we used a likelihood ratio test comparing the full model to a reduced model without the respective predictor. Models analyzing sharing, number of prosocial behaviors in free play, and moment of first inclusion were fit using a Poisson error structure. Models analyzing whether participants included the unfamiliar puppet at all and their decision in the forced-choice trial in the social inclusion task were fit using a binomial error structure. The model for ratio of inclusion was fitted using a Gaussian error structure. We analyzed playing context in the free play with a chi-squared test. All statistical analyses were conducted with R statistical software [42], using the package “lme4” [43] for all GLMMs. All data and script for statistical analyses have been made publicly available via the Open Science Framework (https://osf.io/jasbz/).

## Results

### Pre-analyses

Experimental groups did not differ in age (*F*(2,93) = .030, *p* = .970), gaming duration (*F*(2,45) = 1.730, *p* = .189), and gaming performance (*F*(2,89) = .081, *p* = .922). Dependent variables were not affected by sex (*p*s > .175). Task-order had no significant influence on the dependent variables (*p*s > .095). Although not significant, the effect of task-order on sharing seemed considerable (*χ*^2^(1) = 2.781, *p* = .095), in contrast to all other dependent measures (*p*s > .432). We therefore deviated from our preregistration and decided to include task-order in the null and full model of sharing to control for this potential influence. For all GLMMs, the interaction of age and condition did not reach significance (*p*s > .435).

### Sharing

**Fig 3:**
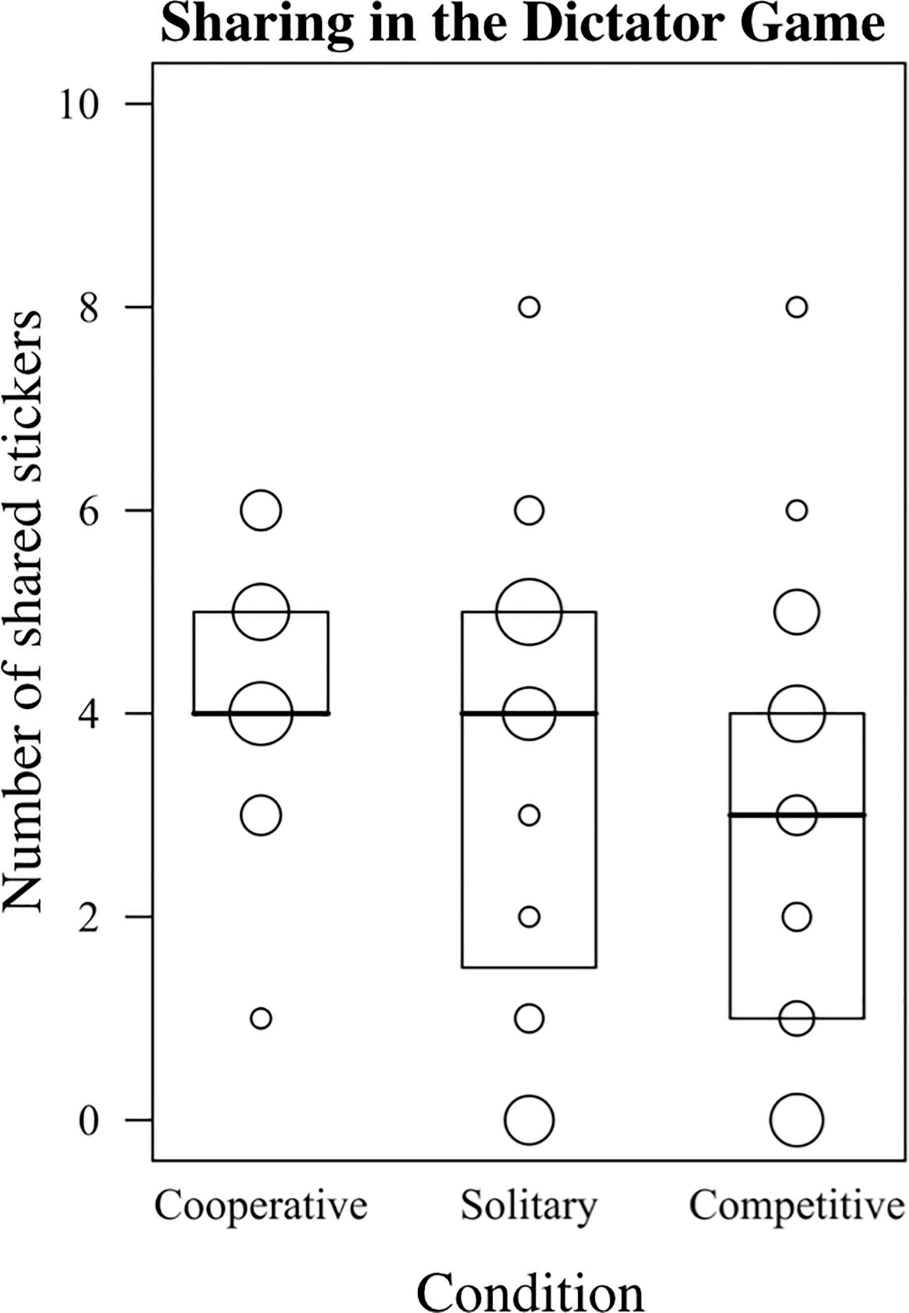
Number of shared stickers by condition. Height of the boxes indicate the interquartile range (IQR) of the sample, solid lines median. Data points are depicted by dots, with larger dots indicating more data points.

Children’s willingness to share was overall significantly influenced by the independent variables (*χ*^2^(6) = 13.177, *p* = .040, Table 1). The full-null model comparison was still significant when conducting the preregistered model with task-order as additional predictor (*χ*^2^(6) = 13.949, *p* = .030, S1 Table). Gaming context affected sharing behavior (*χ*^2^(2) = 6.004, *p* = .050, Fig 3) with higher sharing in the cooperative condition (*M* = 4.33, *SD* = 1.14) than in the competitive condition (*M* = 2.90, *SD* = 2.15, estimate = -.407, *SE* = .166). Sharing in the solitary condition (*M* = 3.55, *SD* = 2.22) did not differ from sharing in the cooperative (estimate = -.203, *SE* = .158) and competitive condition (estimate = .204, *SE* = .165). Although not significant, children tended to share more with increasing age (*χ*^2^(1) = 2.806, *p* = .094, estimate = .116, *SE* = .068). Gaming performance did not affect children’s sharing significantly (*χ*^2^(1) = 1.338, *p* = .247, estimate = .076, *SE* = .066).

**Table 1:** Estimates of the generalized linear mixed model for sharing in the dictator game.

*Coefficient*	*Dictator game*		
	Estimate	SE	p
**Fixed parts**			
(Intercept)	1.526	.144	< .001**
Cooperative vs. Competitive	.407	.166	.014*
Cooperative vs. Solitary	.203	.158	.198
Solitary vs. Competitive	.204	.165	.215
Age	.116	.068	.091^†^
Gaming Result	.076	.066	.246
Task Order	-.169	.115	.141
Sex	-.025	.132	.849
**Random parts**			
τ_00, Dyad_	.051		
N_Dyad_	46		
ICC_Dyad_	.049		
Observations	89		
AIC	384.357		

SE, standard error; ICC, intraclass correlation coefficient; AIC, Akaike information criterion.

^†^*p* < .10

*p <. 05

**p <. 01.

### Social inclusion

Children’s general inclusion, inclusion ratio, and forced-choice were similar across conditions, age, and gaming performances (for descriptive statistics, see Table 2). For these variables, full-null model comparisons did not reach significance (*p*s > .101). Children’s first inclusion was overall significantly influenced by the independent variables (*χ*^*2*^(6) = 23.646, *p* < .001, Table 3). First inclusion was not affected by gaming context (*χ*^2^(2) = 3.751, *p* = .153). Older children (*χ*^2^(1) = 14.722, *p* < .001, estimate = -1.463, *SE* = .412) and children with lower gaming performances (*χ*^2^(1) = 5.287, *p* = .021, estimate = .572, *SE* = .267) included the unfamiliar puppet faster. Importantly, the majority of the children (77.3%) included the unfamiliar puppet immediately with the first pass.

**Table 2:** Descriptive statistics for social inclusion.

	General inclusion	First inclusion	Inclusion ratio	Forced-choice
Condition	*% included*	*M* (*SD*)	*M* (*SD*)	*% to child*
Cooperative	71.88	1.87 (2.14)	.44 (.10)	18.75
Competitive	68.97	2.10 (2.15)	.46 (.14)	31.03
Solitary	74.19	1.39 (1.67)	.46 (.05)	26.67

M, mean; SD, standard deviation. General inclusion refers to whether participants included the unfamiliar puppet within 10 passes or not. First inclusion refers to the pass with which the unfamiliar puppet was included the first time. Inclusion ratio is the ratio between passes to both puppets after inclusion, with a high ratio indicating more passes to the unfamiliar puppet. Forced-choice refers to whether participants wanted the familiar puppet to pass the ball to the unfamiliar puppet or to themselves.

**Table 3:** Estimates of the generalized linear mixed model for first inclusion.

*Coefficient*	*First inclusion*		
	Estimate	SE	p
**Fixed parts**			
(Intercept)	-1.572	.752	.037^*^
Cooperative vs. Competitive	.809	.821	.325
Cooperative vs. Solitary	-1.041	.978	.287
Solitary vs. Competitive	-1.849	1.044	.076^†^
Age	-1.463	.412	< .001^**^
Gaming Result	.572	.267	.032^*^
Sex	-.722	.745	.333
**Random parts**			
τ_00, Dyad_	2.121		
N_Dyad_	43		
ICC_Dyad_	.680		
Observations	66		
AIC	144.554		

SE, standard error; ICC, intraclass correlation coefficient; AIC, Akaike information criterion.

^†^*p* < .10

^*^p < .05

^**^p < .01

### Free play

**Fig 4:**
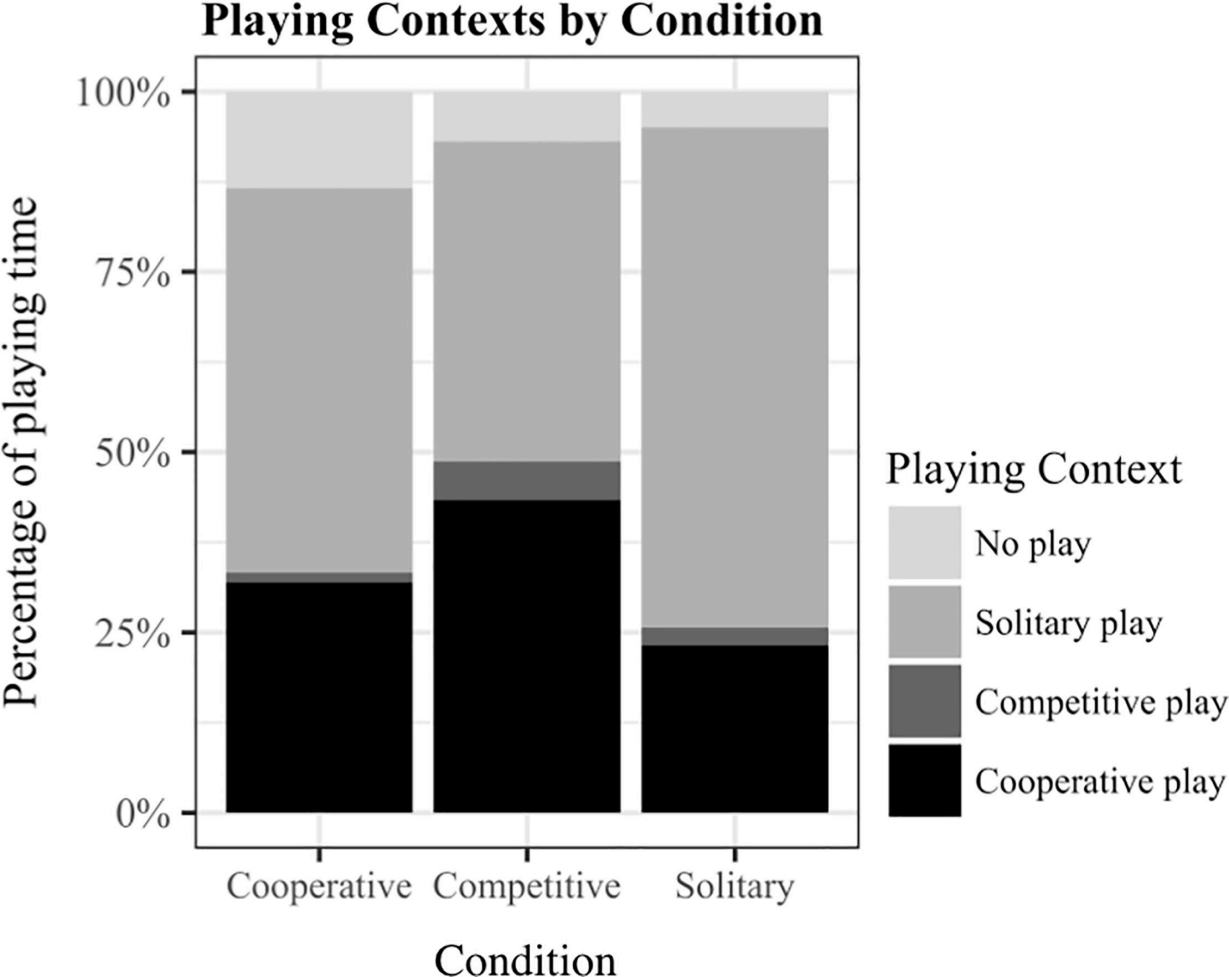
Playing context in free play. Diagram indicates percentage of time played cooperatively, competitively, solitarily and not played in free play by condition.

Prosociality in free play was neither influenced by condition, age, or gaming performance (*χ*^*2*^(6) = 10.329, *p* = .111). Yet, the frequency of prosocial acts was generally low (cooperative condition: *M* = .63, *SD* = .98, competitive condition: *M* = .54, *SD* = 1.07, solitary condition: *M* = .93, *SD* = 1.38). Gaming context did not impact subsequent playing context (*χ*^*2*^(6) = 5.803, *p* = .446). Across conditions, children mostly played cooperatively or solitarily and competitive play occurred rarely (Fig 4).

## Discussion

Our results suggest that gaming contexts (i.e., whether a game is played cooperatively, competitively, or solitarily) affect preschoolers’ prosociality toward uninvolved third-parties. Specifically, children shared more with an unknown peer after playing a cooperative game than after playing a competitive game. However, different gaming contexts did not affect preschoolers’ social inclusion of a third-party and prosociality during free play with the co-player.

The difference in sharing after playing a cooperative and competitive game is in line with the predictions of Social Interdependence Theory (SIT), namely that gaming context has an influence on children’s prosocial behavior [11–13]. However, in contrast to SIT, the sharing rate after playing solitarily did not differ from that after playing cooperatively or competitively. Although, we found the predicted order of sharing rates at the descriptive level, only the difference between the two interdependent conditions reached statistical significance. The small effect size might be explained by the briefness of our intervention, which had a duration of only ten minutes in total. It seems conceivable that cumulative gaming experiences might enhance these differences in sharing rates between gaming contexts. However, this finding supports the assumption that gaming interventions affect subsequent prosociality even after a very brief exposure.

Although we included all three gaming contexts in our design, it still remains unclear whether it is the cooperative or competitive component that mainly drives the difference between these two gaming contexts: Does cooperation increase or competition decrease sharing rates (or do both effects occur at the same time)? Inspection of descriptive results reveals two notable patterns in this regard. First, competitive games seem to lower preschoolers’ willingness to share (*Mdn* = 3) in contrast to cooperative and solitary games (both *Mdn* = 4). Second, low offers (0 to 2 stickers) occurred rarely after playing a cooperative game but more after playing a solitary and competitive game. We propose that, in contrast to competitive and solitary games, cooperative games might promote a sense of fairness [29] rather than generosity. Following this interpretation, our results provide support for both hypotheses: Competitive games might lower prosociality resulting in decreased sharing rates and cooperative games might promote prosociality in the sense of refraining from very low, unfair sharing offers. However, these conclusions are based on descriptive results only and need to be tested in future studies.

In addition, it remains unclear which mechanisms drive the different sharing rates after cooperation and competition. A plausible mechanism might be promoted upstream reciprocity [44]: Being the recipient of a prosocial action might cause more prosociality toward third-parties and trigger a generalized reciprocity. This effect has previously been observed in 4-year-olds [45]. When playing a game cooperatively, children help each other to reach their (shared) goal and this mutual helping elicits benevolence toward others. A converse effect can be assumed for competitive games: Experiencing oppositional behavior of co-players might lower the willingness to act prosocially toward them. In contrast to Leimgruber and colleagues [45], the previously experienced prosocial action in our game (i.e., balancing the marble into a hole) was not the same as in the subsequent prosocial task (i.e., sharing in a dictator game). By using different actions, we demonstrate that the received and transmitted form of prosociality does not necessarily need to be expressed in the same action. Importantly, we do not claim that this effect is limited to children’s games. Experiencing cooperation or competition in non-gaming contexts might have similar effects on children’s prosocial behavior. To clarify whether cooperation induces fairness or benevolence future studies should use measures that more directly assess children’s sense of fairness (e.g., acceptance or rejection of advantages inequity). Additionally, the helpfulness of a cooperative co-player could be manipulated to clarify whether an induced upstream reciprocity is the driving mechanism underlying this effect.

Contrary to our predictions, cooperative games did not increase social inclusion of third-parties compared to competitive or solitary games. Interestingly, age and gaming performance affected the occurrence of children’s first inclusion. Older children tended to include others faster which might be explained by advanced empathy skills [6] or more prior experience with ostracism, which might enable older children to understand a third-party’s desire to join the group more rapidly. Contrary to the promotive effect of positive feelings on prosociality [36], children with lower gaming performances showed a faster inclusion of a third-party player. This is in line with prior findings indicating that negative feelings can actually increase affiliative behavior (e.g., [46]). Thus, the frustration children experienced when losing the game might have fostered subsequent affiliation with third-parties.

Importantly, though, across all three conditions, the frequency of social inclusion was high and social inclusion occurred fast. This ceiling effect might be due to the fact that inclusion was non-costly and the third-party was a neutral unfamiliar other (i.e., not, for example, an outgroup member). Our novel paradigm to assess children’s social inclusion might not have detected potential effects of the different gaming contexts, because of low discriminatory power. Future studies should investigate whether cooperative games foster or competitive games hinder social inclusion of third-parties by changing the present paradigm to provoke lower baseline inclusion rates. This could be achieved by making inclusion costly or difficult. For example, the group membership of the unfamiliar puppet could be manipulated, given that group affiliation has been shown to impact children’s inclusion rates [47].

Surprisingly, our results did not reveal the predicted effect of gaming context on prosociality toward co-players in free play. This finding contrasts with a large body of evidence [15–20,22]. In addition, gaming contexts did not impact subsequent playing contexts. Across all three conditions, children mostly played solitarily or cooperatively. Two reasons might explain the absence of the predicted effects on free play: First, children did not play the KoKo game immediately before the free play task, but engaged in a dictator game or a social inclusion task. The effects of the different gaming contexts might have decreased during this phase before free play started. Similar effects in two subsequent tasks have been observed in children’s social behavior (e.g., [48]). To prevent participants from being overloaded by the study procedure, which already lasted 40 minutes, we decided to skip an additional gaming phase. Moreover, we expected the effects of the game to be the strongest for the free play since children directly interacted with the same co-player from the gaming phases. The second reason refers to the experimental setup. Toy blocks were presented in two separate piles. We decided to conduct a procedure with two piles because we anticipated high frequencies of cooperative play between co-players and wanted to create a high threshold. This, however, might have invited children to engage in solitary play (which we observed most of the time). Further, prosocial behaviors occurred rarely in free play, suggesting that the setting with two piles did not offer many opportunities for helping, sharing, or comforting. Due to floor effects for prosocial behaviors and consequently low discriminatory power, the absence of effect should be interpreted with caution.

For all predicted, but absent effects of gaming context on prosocial behavior (i.e., social inclusion and free play) and playing context, the briefness of our intervention and small sample size need to be considered. This is the first study to systematically examine short-term effects of all three gaming contexts on children’s prosociality. Previous studies investigating these effects used cumulative gaming interventions lasting for at least 3 months and had less well matched non-gaming controls. Additionally, experimenters did not reinforce prosocial or cooperative behaviors in our study design, meaning that the effect is due to the mere gaming experience in the different contexts. As mentioned above, we decided to assess 96 subjects, although a power analysis suggested a sample size of 126 to 133 participants (see preregistration). Our study might be slightly underpowered to detect existing effects on children’s free play.

We found that even a brief gaming experience with peers affects preschoolers’ sharing behavior, highlighting the potential of games to shape social behaviors. Future studies should examine whether different kinds of prosocial behaviors are impacted differently by game-based interventions. For example, some prosocial behaviors (e.g., sharing) might be influenced immediately, while others (e.g., inclusion) only change after a long-term intervention.

Although we addressed several drawbacks of previous studies, our design inevitably had some limitations as well: First, we did not assess baseline performance for dependent measures. Differential effects, such as decreases of prosociality after competition, could be evaluated more clearly with pretests. Future interventional studies might use pretests in order to assess the effects of gaming contexts more accurately. However, it should not be overlooked that sharing could be quite sensitive to repeated assessment in a short period of time. Second, we only examined a sample from a Western, urban background and results might not be generalizable to children from different populations [49]. Cross-cultural comparisons might be a fruitful avenue since games considerably differ between cultures [7], correspond with cultural values [4], and are a crucial nexus in the transmission of cultural values [5]. Additionally, culture shapes the propensity to play games cooperatively or competitively (e.g., [50]) and play can have different functions for child development in different cultural niches [51]. Consequently, enjoyment of cooperative and competitive games might differ between cultural contexts and have different impacts on prosociality. Third, we assessed the impact of simple goal structures, namely cooperative, competitive, and solitary contexts. In everyday life (and especially games) these contexts are not clearly separated and often occur in mixed forms such as cooperation within competition (e.g., two teams playing against each other). SIT does not make clear predictions about behavioral changes of such mixed forms of interdependence. Future studies might address these mixed forms and assess their influence on children’s moral behavior. This could be fruitful in order to explore how robust the effects of cooperation and competition are in the presence of each other. Finally, one should consider that the two types of prosocial behavior towards third-parties (sharing and social inclusion) were directed toward two different recipients (anonymous peer and puppet). Prosocial behavior toward a peer might differ from that toward a puppet operated by the experimenter which might impede a direct comparison of the two variables. However, as described above, psychometrical properties of the social inclusion task generally handicap its interpretation.

## Conclusions

In sum, we find that the way young children engage in games with each other can influence how prosocially they subsequently behave outside of the gaming context. Sharing with an uninvolved third-party systematically differed depending on whether children had previously played a cooperative or a competitive game with a peer. Crucially, this effect occurred immediately after a short amount of playing. Gaming context did not differentially influence overall rates of social inclusion of third-parties or the amount of cooperation in free play with co-players. Our findings lend support to theoretical proposals stating that games provide a fertile ground for young children to experience different modes of interaction which can promote prosocial values. In consideration of the superior number of competitive games in Western societies [52] and common misconceptions about beneficial effects of competition in pedagogy [53], this finding offers interesting implications for preschoolers’ educational environments.

## Supporting information

S1 Table Estimates of the generalized linear mixed model without task-order for sharing in the dictator game. SE, standard error; ICC, intraclass correlation coefficient; AIC, Akaike information criterion.
(DOCX)
