# Supplementary material for: Playing a cooperative game promotes preschoolers’ sharing with third-parties, but not social inclusion
Source: PLoS One. 2019 Aug 19;14(8):e0221092. doi: 10.1371/journal.pone.0221092 (PMC6699707; doi:10.1371/journal.pone.0221092)
Supplement: S1 Table — SE, standard error; ICC, intraclass correlation coefficient; AIC, Akaike information criterion. (DOCX) [file pone.0221092.s001.docx]

**S1 Table. Estimates of the generalized linear mixed model for sharing in the Dictator Game without Task Order.**

| *Coefficient* | *Dictator Game* | | |
| --- | --- | --- | --- |
|  | Estimate | SE | p |
| **Fixed Parts** |  |  |  |
| (Intercept) | 1.436 | .131 | <.001** |
| Cooperative vs. Competitive | .404 | .166 | .015* |
| Cooperative vs. Solitary | .201 | .158 | .204 |
| Solitary vs. Competitive | .203 | .165 | .218 |
| Age | .109 | .068 | .111 |
| Gaming Result | .096 | .065 | .139 |
| Sex | -.018 | .132 | .890 |
| **Random Parts** |  |  |  |
| τ_00, Dyad_ | .052 | | |
| N_Dyad_ | 46 | | |
| ICC_Dyad_ | .049 | | |
| Observations | 89 | | |
| AIC | 384.520 | | |

SE, standard error; ICC, intraclass correlation coefficient; AIC, Akaike information criterion.

^†^*p* < .10, **p* <. 05, ***p* <. 01
